# Metabolic Effects of Acibenzolar-*S*-Methyl for Improving Heat or Drought Stress in Creeping Bentgrass

**DOI:** 10.3389/fpls.2017.01224

**Published:** 2017-07-11

**Authors:** David Jespersen, Jingjin Yu, Bingru Huang

**Affiliations:** ^1^Department of Plant Biology and Pathology, Rutgers University, New Brunswick NJ, United States; ^2^Department of Crop and Soil Sciences, University of Georgia, Griffin GA, United States; ^3^College of Agro-Grassland Science, Nanjing Agricultural University Nanjing, China

**Keywords:** heat, drought, turf, metabolomics, acibenzolar-*S*-methyl

## Abstract

Acibenzolar-*S*-methyl (ASM) is a synthetic functional analog of salicylic acid which can induce systemic acquired resistance in plants, but its effects on abiotic stress tolerance is not well known. The objectives of this study were to examine effects of acibenzolar-*S*-methyl on heat or drought tolerance in creeping bentgrass (*Agrostis stolonifera*) and to determine major ASM-responsive metabolites and proteins associated with enhanced abiotic stress tolerance. Creeping bentgrass plants (cv. ‘Penncross’) were foliarly sprayed with ASM and were exposed to non-stress (20/15°C day/night), heat stress (35/30°C), or drought conditions (by withholding irrigation) in controlled-environment growth chambers. Exogenous ASM treatment resulted in improved heat or drought tolerance, as demonstrated by higher overall turf quality, relative water content, and chlorophyll content compared to the untreated control. Western blotting revealed that ASM application resulted in up-regulation of ATP synthase, HSP-20, PR-3, and Rubisco in plants exposed to heat stress, and greater accumulation of dehydrin in plants exposed to drought stress. Metabolite profiling identified a number of amino acids, organic acids, and sugars which were differentially accumulated between ASM treated and untreated plants under heat or drought stress, including aspartic acid, glycine, citric acid, malic acid, and the sugars glucose, and fructose. Our results suggested that ASM was effective in improving heat or drought tolerance in creeping bentgrass, mainly through enhancing protein synthesis and metabolite accumulation involved in osmotic adjustment, energy metabolism, and stress signaling.

## Introduction

Heat and drought stress are two major abiotic stresses limiting the growth of cool-season perennial grasses. There is increasing use of plant growth regulators (PGRs) in agricultural and horticultural production due to the organic nature and effectiveness of PGRs in promoting plant growth and stress tolerance (Gross and Parthier, 1994; Basra, 2000). However, PGRs vary in their functions of regulating different metabolic processes and in the effectiveness for plant tolerance to different type of stresses (Li et al., 1998; Zhang et al., 2003; Alcázar et al., 2010). Understanding the metabolic processes of specific PGRs involved in regulating plant tolerance to heat or drought stress is important for further knowledge of mechanisms or mode of actions of PGRs and the effective and proper use in improving plant stress tolerance.

Acibenzolar-*S*-methyl (ASM) is an active ingredient in fungicides, which enhances disease resistance by activating the plant’s defense system, which is similar to the functions of salicylic acid (Lawton et al., 1996; Cao et al., 2005; Cavalcanti et al., 2006; Deepak et al., 2006, 2007). Salicylic acid has been found to be involved in both biotic and abiotic stress defense due to its role in signaling for the activation of plant immune systems (Durner et al., 1997; Janda et al., 1999; Horváth et al., 2007). A number of metabolic pathways induced by ASM during pathogen attack, including anti-oxidant metabolism, have also been demonstrated to be activated during abiotic stress (Soylu et al., 2003). Several recent studies under field conditions have demonstrated that ASM improved heat and drought tolerance in creeping bentgrass (Shekoofa et al., 2015; Jespersen and Huang, 2016), and the improved drought performance of creeping bentgrass was associated with ASM-reduction of transpirational water loss (Shekoofa et al., 2015). The mode of action for ASM regulation of abiotic stress tolerance is still unclear and how it alters metabolic pathways to improve stress tolerance is not well understood.

One way to explore metabolic changes is through metabolic profiling, which is a powerful way to quantify and identify metabolites involved in important biological functions for plant responses to abiotic stress (Arbona et al., 2013). The accumulation of major stress responsive metabolites including amino acids, organic acids, sugars, and secondary metabolites is an important process for adaptation to abiotic stresses such as heat or drought (Merewitz et al., 2011; Xu et al., 2013). Alterations in these key metabolites demonstrate not only major shifts in metabolism, but also the activation of important defense mechanisms such as anti-oxidant metabolism and osmotic adjustment (Handa et al., 1983; Reddy et al., 2004). Previous research has found that treatment with ASM has led to increased accumulations of polyphenols and anti-oxidants such as ascorbate and glutathione, which may help protect cells during stress events and improve tolerance to pathogens (Iriti et al., 2005; Kuźniak et al., 2014; Meher et al., 2015). Metabolomic studies have also identified changes in amino acids, organic acids, and sugar accumulations in response to treatment with ASM including glucose, malic acid, and sucrose (Dao et al., 2009; Baldoni et al., 2013).

Changing protein accumulation is also a major part of plant responses to abiotic stresses in order to shift metabolism and activate defense pathways (Kosová et al., 2011). This includes alterations to proteins involved in energy metabolism such as ATP synthase, which is involved in generating the ATP needed for metabolic reactions and providing energy needed for stress defense mechanism, or ribulose-1,6-bisphosphate carboxylase oxygenase (Rubisco), a key enzyme in photosynthesis responsible for carbon fixation (Tezara et al., 1999; Feller et al., 2008). Other protein changes are more directly involved in abiotic stress tolerance mechanisms such as heat shock proteins (HSPs) or dehydrins that play protective roles during stress events (Wang et al., 2004; Hanin et al., 2011). Most changes in protein accumulation due to ASM treatment have been studied in response to pathogen infection and have focused on proteins involved in defense against biotic stresses, finding increased accumulations of pathogenesis related proteins and anti-oxidant proteins such as chitinases, 1,3-betaglucanases, and peroxidases (Burketová et al., 1999; Narusaka et al., 1999; Brisset et al., 2000; Cao and Jiang, 2006). Several proteomic studies have also identified changes to defense proteins such as HSPs, anti-oxidant, and energy proteins, including ATP synthase and Rubisco in response to ASM treatment (Barilli et al., 2012; Gunel et al., 2012; Chen et al., 2015).

The objectives of this study were to determine physiological effects of ASM on heat or drought tolerance in a cool-season perennial grass species, creeping bentgrass and to identify ASM-responsive metabolites which may be responsible for enhanced heat or drought tolerance though metabolic profiling. Furthermore, western blots were used to determine the levels of several key proteins related to abiotic stress tolerance to act as markers to confirm cellular changes in response to ASM treatment.

## Materials and Methods

### Plant Materials

Sod plugs of creeping bentgrass (*Agostis stolonifera* cv. ‘Penncross’) were collected from mature field plots at Rutgers University turfgrass research farm in North Brunswick, NJ, United States. Plants were transplanted into plastic pots (10 cm in diameter, 40 cm deep) which were filled with a 2:1 (v/v) loamy soil and sand mixture. Plants were watered every other day and fertilized weekly with Hoagland’s nutrient solution (Hoagland and Arnon, 1950). Plants were allowed to establish in a greenhouse for 30 days prior to being moved to controlled environment growth chambers (Conviron, Winnipeg, MB, Canada), which were controlled at 20/15°C (day/night), 14-h photoperiod, and photosynthetically active radiation (PAR) of 600 μmol m^-2^ s^-1^.

### Chemical and Stress Treatments

Chemical treatment consisted of ASM applied at a rate of 7.37 mg active ingredient per m^2^ or a water control. Applications were made as a foliar spray which saturated the canopy with a volume of 3.5 ml per pot. Both ASM solution and the untreated control contained 0.05% of the surfactant Tween-20. Chemical treatments began 16 days before the imposition of heat or drought stress and continued on a 14 days interval for the duration of the study. The rate and frequency of treatments were based on common use practices of ASM containing products, when being used for disease protection purposes.

The non-stress control plants were maintained well-watered and received ½ strength Hoagland’s nutrient solution weekly in growth chambers controlled at 20/15°C (day/night). Drought stress was imposed for plants in growth chambers controlled at 20/15°C (day/night) by withholding irrigation for 14 days, at which point soil volumetric water content had dropped to 6%. For heat stress, plants were well-watered and fertilized in growth chamber controlled at 35/30°C (day/night) for 56 days.

Each stress treatment was replicated in four pots and placed in four growth chambers. The ASM treatment and untreated control was replicated in four pots. Plants were relocated within and among growth chambers every 7 days to avoid potential confounding effects of environmental variability in different chambers.

### Physiological Analysis

Several commonly used physiological parameters were evaluated to assess stress tolerance. A visual rating of turf quality (TQ) was used to estimate overall plant health and the level of stress damages on a 1–9 scale, with 1 representing dead plants, and 9 representing completely health plants based on canopy color, density, and uniformity (Krans and Morris, 2007). Relative water content (RWC) was used to measure leaf hydration status according to the methods of Barrs (1962). Approximately 0.2 g of leaf tissue was collected from each pot and immediately the fresh weight was recorded. Leaf tissue was then allowed to become fully turgid by being submerged in de-ionized water for 16 h and 4°C, at which point leafs were gently blotted dry with a paper towel and the turgid weight of the rehydrated leafs was recorded. Leaf tissue was then placed in an envelope and dried in an 80°C oven for at least 72 h, afterward leaf dry weight was recorded for each sample. RWC was calculated using the equation RWC = (Fresh weight – Dry weight)/(Turgid weight – Dry weight) × 100%. Chlorophyll content (CHL) was measured to estimate levels of leaf senescence and overall photosynthetic health based on the procedures of Hiscox and Israelstam (1979). Approximately 0.1 g of fresh leaf tissue was place into 10 ml of dimethyl sulfoxide and allowed to incubate for 5 days in completely darkness to extract pigments from the leaf tissue. The resulting solution was measured using a spectrophotometer (Genesys 2, Spectronic Instruments, Inc., Rochester, NY, United States) at 663 and 645 nm. Leaf tissue was then filtered from the remaining solution and dried in an 80°C oven for at least 72 h to obtain leaf dry weights. Chlorophyll content was calculated on a dry weight basis using the equations described by Arnon (1949). Measurements to assess plant health were performed every 7 days for the duration of the experiments. Additionally, representative leaf tissue samples were harvested at every sampling day and flash frozen in liquid nitrogen and stored at -80°C for further analysis.

### Western Blotting Analysis of Protein Expression

Western blotting was performed to estimate differences in protein levels related to energy metabolism and stress defense induced by stress or chemical treatments. Tissues collected at 56 days of heat stress or 14 days of drought stress, when the greatest physiological differences between treatments was found, were used for western blotting. Proteins were extracted by grinding tissue with liquid nitrogen and then extracting proteins using a tris-LDS extraction buffer. After 30 min of sonication samples were centrifuges of 5 min at 10,000 × *g*. Protein content in the supernatant was quantified using the Bradford assay (Bradford, 1976) and stored at -80 C for further analysis.

A 20 μg of protein per lane was loaded onto a Novex Bis-Tris gel, and run at 200 volts for 30 min using a XCell SureLock Mini-Cell (Thermo Fisher, Waltham, MA, United States). Proteins were then transferred to a nitrocellulose membrane with a blot module run at 25 volts for 90 min. Membranes were then washed with de-ionized water for 5 min two times. Membranes were then blocked using a TBS-T buffer which contained 20 mM Tris-Base, 137 mM NaCl, 2% low-fat milk powder, 0.1% Tween 20, pH 7.6 by incubating on a shaker for 1 h. The blocking solution was then discarded and membranes were incubated for 16 h at 4°C in an identical TBS-T buffer which contained the desired dilution of the primary antibody. The membrane was then washed in a TBS-T buffer which excluded the blocking agent (milk powder) for 5 min repeatedly for a total of five washes. The membrane was then incubated for 30 min in the TBS-T buffer which now contains the desired dilution of the secondary antibody. Following this the membranes were washed with DI water, and the ELC HRP chemiluminesent substrate was used to determine protein levels using a ccd imaging system (Bio-Rad, Hercules, CA, United States). ImageJ was used to quantify relative protein band intensities compared to actin control bands. Proteins quantified include ATP synthase, ribulose-1,5-bisphosphate carboxylase oxygenase (Rubisco), heat shock protein 20 (HSP-20), dehydrin, PR-3, and actin using commercially prepared antibodies (Agrisera, Vännäs, Sweden).

### Metabolite Analysis

Metabolite analysis was performing using gas chromatography mass spectrometry to better understand changes in metabolism caused by environmental or chemical treatments following the procedures but forth by Du et al. (2011). Leaf tissues collected at 56 days of heat stress or 14 days of drought stress, when the greatest physiological differences between treatments was found, were used for metabolomic analysis. Extraction was performed according to Roessner et al. (2000) with modifications. Frozen samples were first lyophilized (FreeZone 4.5 Labconco, Kansas City, MO, United States) and ground to a fine powder. A total of 25 mg of leaf powder per sample was extracted in 1.4 ml of 80% (v/v) aqueous methanol at ambient temperature under 200 rpm for 2 h. Ribitol (10 μl of a 2 mg ml^-1^ solution) was added to each sample as an internal standard prior to incubation. Samples were then extracted for 15 min in a 70°C water bath, and centrifuged at 12,000 rpm for 30 min. The resulting supernatant were transferred to new tubes and 1.4 ml of water and 0.75 ml of chloroform were added. Samples were thoroughly vortexed, centrifuged for 5 min at 5,000 rpm, and 2 ml of the polar phase (methanol/water) was decanted into 1.5 ml HPLC vials which were then dried in a benchtop centrifugal concentrator (Centrivap Labconco, Kansas City, MO, United States). The dried polar phase was methoximated with 80 μl of methoxyamine hydrochloride (20 mg ml^-1^) at 30°C for 90 min and was trimethylsilylated with 80 μl *N*-Methyl-*N*-trimethylsilyltri-fluoroacetamide (with 1% trimethylchlorosilane) for 60 min at 70°C.

GC-MS analysis followed the procedure described in Qiu et al. (2007). The derivatized extracts were analyzed with a PerkinElmer gas chromatograph coupled with a TurboMass-Autosystem XL mass spectrometer (PerkinElmer Inc., United States). Extract aliquots of 1 μl were injected into a DB-5MS capillary column (30 m × 0.25 mm × 0.25 μm, Agilent J&W Scientific, Folsom, CA, United States). The inlet temperature was set at 260°C. After a 6.5 min solvent delay, initial GC oven temperature was set at 60°C; 1 min after injection, the GC oven temperature was raised 5°C min^-1^, and finally held at 280°C for 15 min. The injection temperature was set to 280°C and the ion source temperature was set to 200°C. The helium carrier gas had a constant flow rate of 1 ml min^-1^. Measurements were made with electron impact ionization (70 eV) in the full scan mode (m/z 30–550). Turbomass 4.1.1 software (PerkinElmer Inc., United States) coupled with commercially available compound libraries (NIST 2005, Wiley 7.0) was used to identify the detected metabolites. For GC/MS results, compounds were identified based on retention times and comparison with reference spectra in mass spectral libraries. Peaks areas of metabolites were integrated with the Genesis algorithm, and relative quantities were calculated using the internal ribitol standard.

### Statistical Analysis

Treatment effects were analyzed using two-way ANOVA and means were separated using Fishers LSD. Additionally, metabolite data was analyzed using principle component analysis (PCA) using JMP Pro (v. 12, SAS Institute Inc., Cary, NC, United States). The heat map was generated using R (v. 3.4) and the package “heatmap”; representing fold changes from non-stress treatments using a log2 transformation to assist in visualization of the range of data. MetaboAnalyst (v. 3.0) was used to perform pathway enrichment and topology analysis (Xia and Wishart, 2016). Metabolic pathways with a Holm adjusted *p*-value of <0.05 were considered to be significantly altered by treatment. A Venn diagram comparing metabolites significantly altered by ASM treatment was created using VennPlex (Cai et al., 2013).

## Results

### Physiological Improvement of Heat or Drought Tolerance As Affected by ASM

**FIGURE 1:**
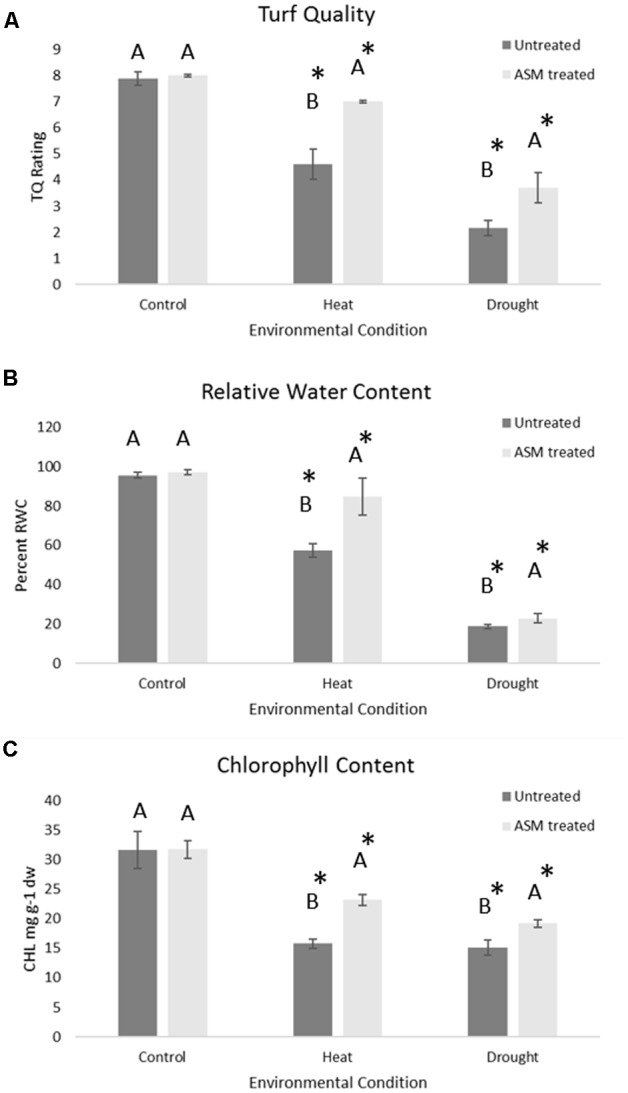
Physiological responses of **(A)** turf quality, **(B)** relative water content, and **(C)** chlorophyll content in bentgrass under non-stress conditions, 56 days heat stress, or 14 days drought stress when treated with ASM or in untreated controls. Bars represent standard error, letters are LSD groupings, with different letters being significant at *p* < 0.05 for treatments in a given environment (control, heat, or drought), while an asterisks (^∗^) indicates a significant difference for a given treatment when compared to non-stress control conditions.

Under non-stress conditions, no significant differences were found between ASM and untreated control plants for TQ, RWC, or CHL (**Figure 1**). Under heat or drought stress, TQ, RWC, and CHL were significantly lower in both ASM treated and untreated control plants compared to the non-stress control. Plants treated with ASM maintained significantly higher TQ (7.0), RWC (84.7%), and CHL (23.2 mg g^-1^ dw) than those of the untreated control plants with TQ of 4.6, RWC of 57.5%, and CHL of 15.8 mg g^-1^ dw at 56 days of heat stress (**Figure 1**). During drought stress plants treated with ASM maintained significantly higher TQ (3.7), RWC (24.5%), and CHL (19.2 mg g^-1^ dw) compared to untreated control plants with TQ of 2.1, RWC of 18.7%, and CHL of 15.1 mg g^-1^ dw, at 14 days drought. These significant physiological differences were only present at the end of heat stress at 56 days, or end of drought stress at 14 days when the greatest amount of stress damages had been accumulated.

### Alteration of Protein Expression by ASM under Heat or Drought Stress

**FIGURE 2:**
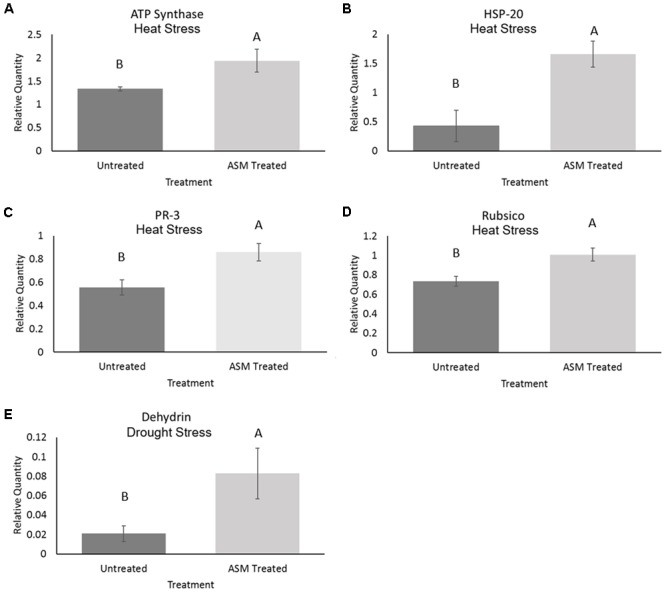
Protein content of **(A)** ATP synthase, **(B)** heat shock protein-20, **(C)** PR-3, and **(D)** Rubisco in ASM treated and untreated control plants at 56 days heat stress, and **(E)** dehydrin content in ASM treated and untreated plants at 14 days drought stress. Values are presented as relative quantities compared to actin controls. Bars represent standard error, letters are LSD groupings with treatments with different letters being significant at *p* < 0.05.

The expression level of selected proteins known to play positive roles in plant tolerance to heat or drought stress was examined in ASM treated and untreated plants to determine whether ASM improved heat or drought tolerance by ASM may be associated with the alteration of the expression of those stress-related proteins. Under control conditions there were no significant differences in the relative protein quantities for ATP synthase, Rubisco, PR-3 or HSP20. Under heat stress conditions ASM treatment resulted in 1.4-fold increases in both the abundance of ATP synthase and Rubisco, 1-5-fold in PR-3 proteins and 3.9-fold in HSP20 compared to the untreated plants (**Figure 2**). No significant differences were found in dehydrin levels between ASM treated and untreated plants exposed to heat stress. Under drought stress ASM treatment lead to a significant increase in the accumulation of dehydrin with a nearly 5.4-fold greater protein abundance compared to the untreated control plants (**Figure 2**). ASM had no significant effects on the expression of other proteins under drought stress.

### Alteration of Metabolite Accumulation by ASM under Heat or Drought Stress

**FIGURE 3:**
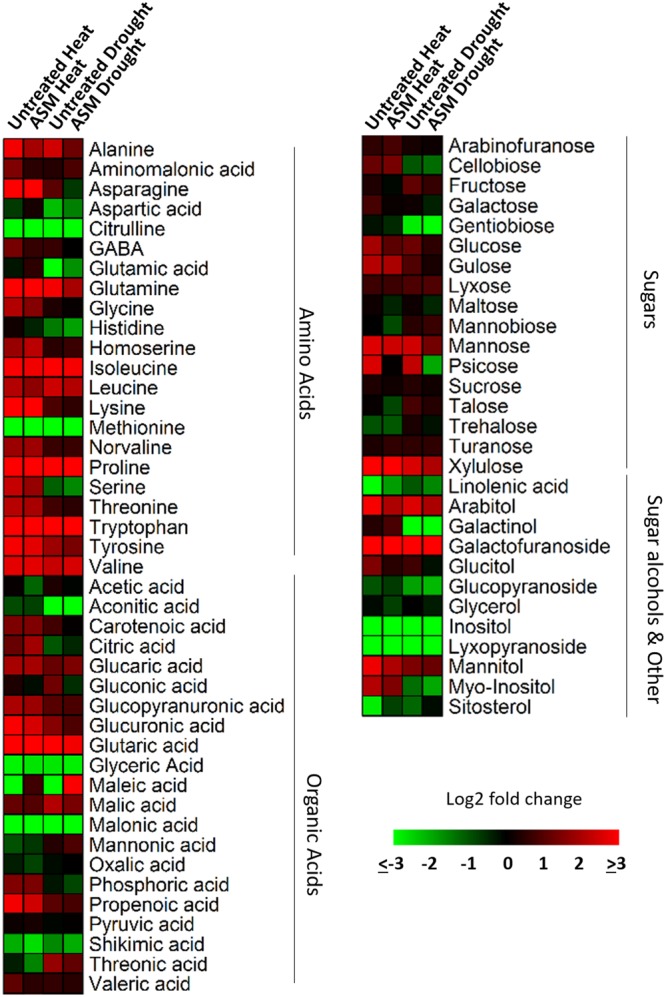
Heat map showing fold changes for 72 identified metabolites in plants exposed to 56 days heat stress or 14 days drought stress and treated with ASM and untreated controls. Fold changes are made in comparison to non-stress condition plants with red representing up-regulation and green representing down-regulation.

A total of 72 metabolites were identified and quantified using GC/MS, including 22 amino acids, 21 organic acids, 17 sugars, and 12 other metabolites which included sugar alcohols, glycosides, fatty acids, and phytosterols (**Table 1**). Of the 72 metabolites, 49 metabolites (21 amino acids, 10 organic acids, 12 sugars, and 6 other metabolites) showed significantly higher content and 17 metabolites (8 organic acids, 4 sugars, and 5 other metabolites) had lower content in heat-stressed plants than the non-stress control plants with or without ASM treatment (**Figure 3**). Drought-stressed plants had greater contents of 33 metabolites (10 amino acids, 12 organic acids, 9 sugars, and 2 other metabolites) and lower contents of 22 metabolites (5 amino acids, 7 organic acids, 4 sugars, and 6 other metabolites) compared to the non-stress control plants (Supplementary Table S1).

**Table 1:** Metabolites identified by GC-MS.

	Metabolite	m/z	RT
1	Glycine	58	7.09
2	Pyruvic acid	117	8.89
3	Acetic acid	147	9.33
4	Propenoic acid	147	9.62
5	Alanine	116	10.05
6	Oxalic acid	147	11.06
7	Phosphoric acid	133	12.05
8	Valine	218	13.14
9	Leucine	158	14.70
10	Glycerol	205	14.80
11	Isoleucine	158	15.28
12	Proline	143	15.36
13	Glyceric acid	189	16.28
14	Serine	204	17.09
15	Threonine	219	17.75
16	Maleic acid	142	18.17
17	Norvaline	175	18.81
18	Homoserine	218	19.39
19	Aminomalonic acid	218	19.87
20	Malic acid	147	20.38
21	Valeric acid	143	20.83
22	Methionine	177	21.04
23	Aspartic acid	218	21.13
24	GABA	175	21.33
25	Threonic acid	205	22.10
26	Xylulose	205	22.44
27	Glutaric acid	129	22.46
28	Lyxose	204	23.25
29	Glutamic acid	246	23.50
30	Asparagine	116	24.63
31	Aconitic acid	147	26.41
32	Arabinofuranose	217	26.62
33	Glutamine	157	26.89
34	Shikimic acid	204	27.73
35	Glucaric acid	117	27.78
36	Citric acid	273	27.85
37	Citrulline	74	27.95
38	Fructose	217	29.12
39	Psicose	306	29.26
40	Galactose	319	29.28
41	Talose	205	29.42
42	Glucose	205	29.77
43	Histidine	155	29.80
44	Lysine	175	29.93
45	Mannitol	205	30.05
46	Tyrosine	218	30.22
47	Mannobiose	217	30.60
48	Arabitol	103	30.95
49	Gluconic acid	205	31.24
50	Malonic acid	205	31.87
51	Inositol	191	31.94
52	Myo-inositol	217	33.09
53	Glucitol	205	33.51
54	Tryptophan	202	35.26
55	Linolenic acid	79	35.43
56	Mannose	204	36.03
57	Carotenoic acid	133	36.44
58	Glucuronic acid	217	38.11
59	Galactofuranoside	105	39.97
60	Sucrose	437	42.22
61	Turanose	147	42.25
62	Gulose	204	42.67
63	Cellobiose	204	43.45
64	Maltose	204	43.59
65	Mannonic acid	217	43.85
66	Lyxopyranoside	204	44.45
67	Trehalose	217	44.59
68	Glucopyranuronic acid	105	46.25
69	Galactinol	204	46.99
70	Gentiobiose	204	48.91
71	Sitosterol	129	51.71
72	Glucopyranoside	217	52.86

*List of 79 identified metabolites and associated m/z scores (mass to charge ratios) and retention times in minutes (RT).*

**FIGURE 4:**
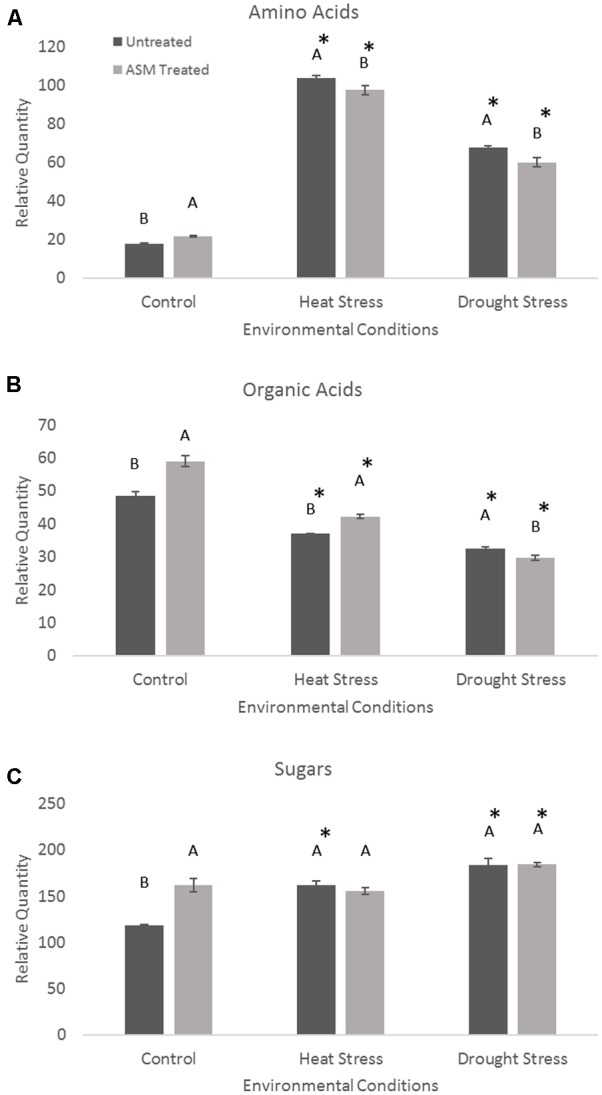
Total relative contents for the major metabolite groups of **(A)** amino acids, **(B)** organic acids, and **(C)** sugars when treated with ASM or untreated and exposed to non-stress control, heat stress, or drought stress conditions. Bars represent standard error, letters are LSD groupings, with different letters being significant at *p* < 0.05 for treatments in a given environment (control, heat, or drought), while an asterisks (^∗^) indicates a significant difference for a given treatment when compared to non-stress control conditions.

Exogenous applications of ASM resulted in significant changes in metabolite levels during heat or drought stress conditions (**Figure 4**). The content totals of amino acids significantly increased during heat stress, with untreated plant having a 485% increase, resulting in significantly higher amino acid totals than ASM treated plants which increased 348% from non-stress control conditions. Contrastingly, organic acid totals of the measured metabolites decreased in both ASM treated and untreated plants during heat stress. Under heat stress ASM treated plants had significantly more organic acid content than untreated plants, with ASM treated plants’ organic acid totals being 71% of control conditions, and untreated plants’ organic acid totals being 77% of untreated plants under control conditions. Total sugar metabolite contents increased 37% in untreated plant during heat stress, but were not significantly changed in ASM treated plants. Since ASM treated plants had higher sugar content under non-stress control conditions, the increase in untreated plants resulted in no significant differences in total sugar content between ASM and untreated plants during heat stress. During drought stress, total amino acid content increased in both ASM treated (175%) and untreated plants (281%) compared to control conditions, but to a significantly greater degree in untreated plants. Organic acid totals declined during drought stress but to a significantly greater degree in ASM treated plants (50% of controls), compared to untreated plants (67% of controls). Total sugar content significantly increased in ASM treated and untreated plant during drought stress, by 14 and 55%, respectively. However, there were no significant differences in total sugar content between two treatments during drought.

**FIGURE 5:**
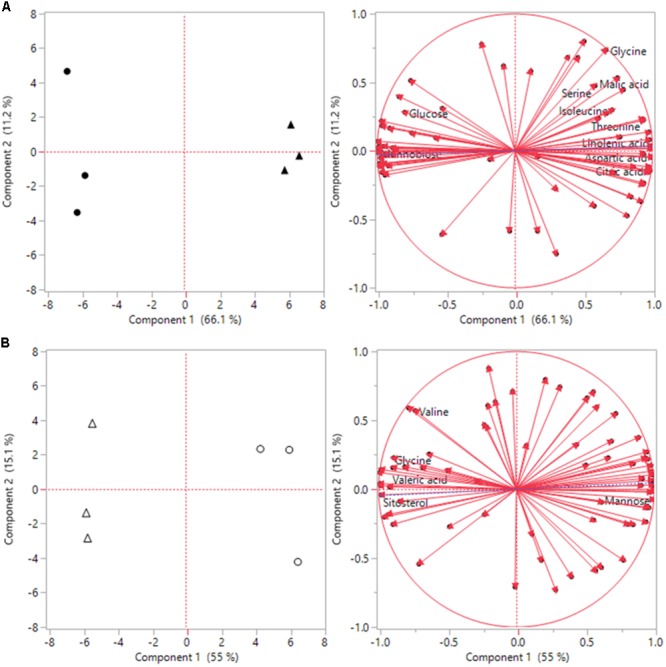
Principle component analysis (PCA) score plots and loading plots for the first two components for **(A)** heat, and **(B)** drought stress conditions. Solid black circles represent untreated plants during heat stress, black triangles represent ASM treated plants during heat stress. White circles represent untreated plants during drought stress, and white triangles represent ASM treated plants under drought stress.

Under heat stress conditions 46 metabolites where significantly altered out of the 72 identified metabolites when comparing ASM to untreated plants. This included 15 amino acids, of which 8 had significant increases and 7 had decreases when compared to untreated plants (**Table 2**). Amino acids with higher contents in ASM treated plants were aspartic acid, glutamic acid, glutamine, glycine, isoleucine, lysine, serine, and threonine; lower content amino acids were alanine, aminomalonic acid, asparagine, GABA, histidine, proline, and tryptophan. A total of 13 organic acids were found to have differential accumulation with 6 having higher content and 7 having lower content compared to untreated plants at 54 days heat stress. Organic acids with greater accumulations included aconitic acid, carotenoic acid, citric acid, gluconic acid, malic acid, and phosphoric acid; organic acids with lower accumulations in ASM treated plants included acetic acid, glucaric acid, oxalic acid, propenoic acid, pyruvic acid, threonic acid, and valeric acid. Metabolite analysis identified 8 sugars that had significant differences, with 3 being higher and 5 being lower, as well as 10 sugar alcohols and other metabolites in which 5 were increased and 5 were decreased in ASM treated compared to untreated plant during heat stress. Sugars that had higher content in ASM treated plants during heat stress included arabinofuranose, gentiobiose, and gulose; sugars that were significantly lower in content included fructose, galactose, glucose, mannobiose, and talose. Other metabolites which were differentially accumulated include galactinol, galactofuranoside, glucopyranoside, linolenic acid, and sitosterol which had increased accumulations; while arabitol, glucitol, glycerol, inositol, and mannitol had decreased accumulations when comparing ASM treated to untreated plants during heat stress. PCA analysis results found component 1 accounted for 66.1% of the variance and component 2 accounted for 11.2% (**Figure 5**). ASM treated and untreated groups are distrinctly separated on component 1, metabolites with high loading factors (top 15%) for component 1 included aspartic acid, sitosterol, linolenic acid, fructose, glutamic acid, and citric acid.

**Table 2:** Quantities of select metabolites affected by Acibenzolar-*S*-methyl (ASM) treatment during heat or drought stress.

	Non-stress control			Non-stress ASM			Heat stress control			Heat stress ASM			Drought stress control			Drought stress ASM		
Metabolite	Rel. Quant	*SE*	LSD	Rel. Quant	*SE*	LSD	Rel. Quant	*SE*	LSD	Rel. Quant	*SE*	LSD	Rel. Quant	*SE*	LSD	Rel. Quant	*SE*	LSD
Aspartic acid	0.581	0.016	A	0.482	0.006	B	0.358	0.008	B^∗^	0.580	0.014	A^∗^	0.124	0.001	B^∗^	0.168	0.002	A^∗^
Carotenoic acid	0.015	0.000	B	0.020	0.000	A	0.042	0.001	B^∗^	0.056	0.002	A^∗^	0.026	0.001	A^∗^	0.021	0.001	B
Citric acid	1.374	0.021	A	1.101	0.039	B	3.193	0.086	B^∗^	4.105	0.091	A^∗^	0.684	0.011	A^∗^	0.811	0.012	A^∗^
Fructose	23.115	0.468	B	33.678	1.521	A	30.067	0.587	A^∗^	19.304	0.220	B^∗^	50.769	0.966	A^∗^	52.311	2.171	A^∗^
Glutamic acid	5.602	0.166	A	4.115	0.057	B	4.686	0.147	B^∗^	5.982	0.050	A^∗^	0.706	0.039	B^∗^	1.279	0.027	A^∗^
Glucose	4.078	0.223	B	6.587	0.434	A	15.495	0.654	A^∗^	13.883	0.358	B^∗^	9.908	0.086	A^∗^	9.495	0.103	A^∗^
Glycine	2.802	0.132	B	4.411	0.064	A^∗^	11.911	0.550	B^∗^	13.186	0.211	A	3.596	0.190	B^∗^	4.688	0.196	A
Linolenic acid	0.329	0.007	B	0.449	0.014	A	0.031	0.001	B^∗^	0.124	0.007	A^∗^	0.160	0.004	A^∗^	0.150	0.002	A^∗^
Malic acid	4.328	0.178	B	5.515	0.132	A	9.907	0.190	B^∗^	10.774	0.183	A^∗^	18.545	0.114	A^∗^	14.588	0.102	B^∗^
Mannobiose	0.263	0.016	A	0.313	0.004	A	0.262	0.005	A	0.172	0.003	B^∗^	0.368	0.013	B^∗^	0.502	0.003	A^∗^
Mannonic acid	0.113	0.006	A	0.108	0.003	A	0.058	0.002	A^∗^	0.069	0.002	A^∗^	0.152	0.002	B^∗^	0.204	0.002	A^∗^
Serine	2.840	0.054	B	4.559	0.292	A	14.117	0.693	B^∗^	15.329	0.290	A^∗^	1.306	0.008	A^∗^	1.486	0.038	A^∗^
Sitosterol	0.604	0.005	A	0.580	0.007	A	0.086	0.002	B^∗^	0.339	0.018	A^∗^	0.261	0.006	B^∗^	0.527	0.011	A^∗^
Valeric acid	0.049	0.002	B	0.066	0.001	A	0.105	0.003	A^∗^	0.094	0.005	B^∗^	0.074	0.001	B^∗^	0.091	0.002	A^∗^
Valine	0.270	0.019	A	0.282	0.009	A	1.757	0.030	A^∗^	1.816	0.040	A^∗^	1.358	0.045	B^∗^	1.628	0.097	A^∗^

*Differences in capital letters indicate a significant difference between Control and ASM treatments under a given environmental stress condition, and ^∗^ indicates a significant difference between stress and non-stress conditions for a given treatment. Rel. Quant, Relative quantities; *SE*, Standard error; LSD, LSD grouping at *p* < 0.05.*

Under drought stress conditions 24 metabolites were significantly different between ASM and untreated plants. Of the 9 amino acids found to be significantly different, 5 had higher content and 4 had lower content under drought when compared to untreated plants (**Table 2**). Amino acids with higher content were aminomalonic acid, aspartic acid, glutamic acid, glycine, and valine; amino acids with lower content were alanine, GABA, proline, and tryptophan. Out of 8 differentially accumulated organic acids, 3 had higher content, aconitic acid, mannonic acid, and valeric acid, while 5, carotenoic acid, gluconic acid, maleic acid, malic acid, and threonic acid, had lower content in ASM treated plants during drought when compared to untreated plants. Also during drought 4 sugars and 3 sugar alcohols and other metabolites showed significant differences with 1 sugar having higher content and 3 having lower content, and 1 other metabolite having higher content and 2 having lower content when comparing ASM treated to untreated plants at 14 days drought stress. Sugars with greater accumulations in ASM treated compared to untreated plants during drought included mannobiose, while lower accumulations were found with gulose, mannose, and psicose. Additional the metabolite sitosterol had higher accumulation, while inositol and lyxopyranoside had lower accumulation with ASM treatment during drought. PCA analysis results found component 1 accounted for 55.0% of the variance and component 2 accounted for 15.1% (**Figure 5**) during drought stress. ASM treated and untreated groups are separated along component. Metabolites with high loading (top 15%) factors for component 1 included inositol, mannose, malic acid, alanine, citric acid, asparagine, and gluconic acid.

**FIGURE 6:**
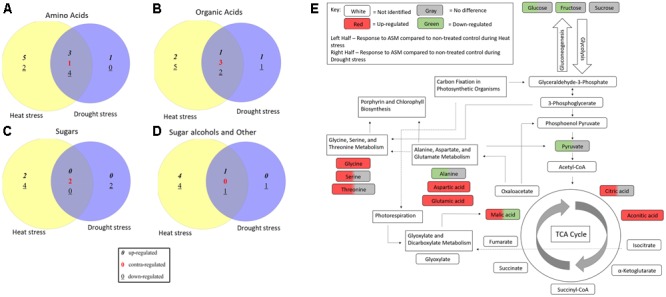
Venn diagrams comparing **(A)** amino acids, **(B)** organic acids, **(C)** sugars, and **(D)** sugar alcohols and other metabolites significantly altered by ASM treatment compared to untreated plants under non-stress, heat, and drought stress conditions. **(E)** Visualization of metabolic pathways significantly altered during heat or drought stress when treated with ASM and relationships between these pathways based on enrichment analysis with Metaboanalyst and KEGG maps.

Acibenzolar-*S*-methyl treatment cause significant changes in metabolites levels compared to untreated planted during both heat and drought stress treatments, although these changes were not always common between the two stresses (**Figure 6**). Pathway analysis identified 18 metabolic pathways that were significant enriched using Holm’s adjusted *p*-value when comparing ASM treated and untreated plants under heat stress. While under drought stress conditions, 11 pathways were found to be significantly altered when comparing ASM treated and untreated plants. A total of nine pathways were commonly identified in both stresses and include alanine, aspartate and glutamate metabolism, glyoxylate and dicarboxylate metabolism, aminoacyl-tRNA biosynthesis, arginine and proline metabolism, citrate cycle (TCA cycle), carbon fixation in photosynthetic organisms, beta-alanine metabolism, nicotinate and nicotinamide metabolism, and porphyrin and chlorophyll metabolism. Additional pathways affected by ASM treatment during abiotic stress include glycine, serine, and threonine metabolism during drought, and glycerolipid metabolism, nitrogen metabolism, and glycolysis and gluconeogenesis during heat stress. Many of these pathways are interrelated and may result in widespread changes in plant metabolism.

## Discussion

Both abiotic stresses of heat or drought resulted in damage to plants over the course of the 54-days heat stress period, or 14-days drought period. However, plants which received ASM treatment had increased stress tolerance as demonstrated by lesser declines in overall visual TQ, leaf hydration status as measured by RWC, and CHL compared to untreated plants. These three measurements of TQ, RWC, and CHL are frequently used to assess abiotic stress tolerance and have previously been shown to decline in turfgrasses as abiotic stress damages accumulate, with more tolerant cultivars maintaining high levels of these parameters than sensitive cultivars (Jespersen et al., 2013). The physiological analysis demonstrates that treatment with ASM was effective in improving creeping bentgrass tolerance to both drought and heat stress.

The ability of ASM to prime a plant’s defense mechanisms to protect against disease has been widely documented (Latunde-Dada and Lucas, 2001; Maxson-Stein et al., 2002; Soylu et al., 2003; Beckers and Conrath, 2007), but how treatment with ASM may enhance abiotic stress tolerance has not been thoroughly studied. Plant defenses primed via ASM application induces signaling in the salicylic acid pathway, which has previously been shown to influence abiotic stress tolerance (Singh and Usha, 2003; Larkindale and Huang, 2005; Czovek et al., 2006). Plants treated with ASM have increased activities of anti-oxidant enzymes such as catalase and superoxide dismutase (Soylu et al., 2003; Deepak et al., 2006) which also have increased activity with application of salicylic acid (He et al., 2003) as an important factor influencing the damage caused by reactive oxygen species during stress events. The remaining discussion will focus on whether the positive effects of ASM on heat or drought tolerance in creeping bentgrass were associated with alteration of proteins and metabolites.

### ASM-Responsive Proteins and Metabolites Related to Heat Tolerance

Induction of stress-protective proteins or energy-related proteins are critically important plant adaption to heat stress (Wahid et al., 2007). The proteins ATP synthase, HSP-20, PR-3, and Rubisco were all found via western blotting to have significantly higher accumulations in ASM treated plants compared to untreated plants during heat stress conditions. ATP synthase and Rubisco are both important proteins involved in energy metabolism that have previously been shown to be differentially accumulated during heat stress in bentgrasses (Xu and Huang, 2010; Jespersen and Huang, 2015). Maintaining these proteins may allow the plant to continue to generate energy via photosynthesis and utilize energy for stress defense and repair mechanisms, as carbon starvation is a common occurrence during prolonged heat stress (Wahid et al., 2007). HSPs, including HSP-20, are important chaperones which can refold proteins damages by heat stress and have been found to have higher accumulations in more heat tolerance turfgrasses (Xu et al., 2011). The higher levels of HSP-20 in ASM treated plants may indicate a greater stress response responsible for stabilizing protein metabolism during high temperature events. Although PR-3, a chitinase, has not been implicated in abiotic stress tolerance it is an important pathogen defense protein which has been demonstrated to be activated by salicylic acid signaling and likewise by applications of ASM (Mandal et al., 2008). This may indicate that some of the pathways being activated during heat stress are commonly activated during other challenges to the plant as well.

Amino acids are important building blocks that feed into many metabolic processes and are involved in nitrogen metabolism, protein synthesis, energy relations and signaling functions (Staswick and Tiryaki, 2004; Meister, 2012). ASM treatment resulted in significantly increased accumulations of the amino acids aspartic acid, glutamic acid, glutamine, glycine, isoleucine, lysine, serine, and threonine during heat stress compared to untreated plants. Increased accumulations of the amino acids aspartic acid, glutamic acid, glycine, and serine have previously been associated with increased levels of stress tolerance in turfgrasses (Jespersen et al., 2015; Li et al., 2016). The higher accumulation of these amino acids in ASM treated plants may be part of major shifts in metabolism and activation of defense pathways. Several of these amino acids including aspartic acid, isoleucine, and threonine can act as major precursors to other amino acids and metabolites which may represent a shift in metabolism in response to stress (Azevedo et al., 2006; Joshi et al., 2010), as preferential accumulation of specific amino acids is a documented response to heat stress as plants respond to elevated temperatures (Mayer et al., 1990). Glutamic acid is an important precursor to chlorophyll biosynthesis (Forde and Lea, 2007). Elevated levels of glutamic acid is a potentially a factor contributing to higher chlorophyll levels observed during heat stress in ASM treated plants by helping to maintain chlorophyll synthesis. The amino acid glycine can function as a compatible solute to help project the cell during stress events (Yancey et al., 1982). Furthermore, glycine is involved in the synthesis of glutathione, which is an important component of anti-oxidant metabolism (Noctor et al., 1997). Elevated levels of glycine may help ASM treated plants undergo osmotic adjustment and enhance anti-oxidant metabolism to protect against heat related damages. The activation of anti-oxidant pathways has previously been shown to be an effect of ASM treatment in plants, enhancing defense pathways (Brisset et al., 2000; Kuźniak et al., 2014). During heat stress Rubisco increasingly binds to oxygen instead of carbon dioxide in a process known as photorespiration (Wingler et al., 2000). Both glycine and serine are involved in the photorespiritory cycle which has been associated with improved stress tolerance (Moreno et al., 2005). Higher levels of glycine and serine found in ASM treated plants may be an important aspect of regulating photorespiration during heat stress to maintain an active photosynthetic system.

Organic acids have many important metabolic functions including being intermediates in the citric acid cycle, as intermediates in nitrogen metabolism, involvement in redox balance, and can be accumulated to act as compatible solutes (Sweetlove et al., 2010). Organic acids have previously been shown to be responsive to heat stress in a number of species, including in model species such as *Arabidopsis thaliana* and in turf species such as *A. stolonifera* and *Festuca arundinacea* (Kaplan et al., 2004; Yu et al., 2012; Xu et al., 2013). The metabolites aconitic acid, carotenoic acid, citric acid, gluconic acid, malic acid, and phosphoric acid had significantly greater accumulations in ASM treated plants than untreated plants during heat stress, and may help explain the different levels of heat tolerance seen in the two treatments. Carotenoic acids and carotenoids have been widely implicated in stress tolerance mechanisms by acting to help absorb excess energy and prevent the creation of ROS while helping maintain thylakoid integrity (Havaux, 1998). Higher accumulations of carotenoic acids in ASM treated plants may aid preventing damage to photosynthetic machinery during heat stress. Citric acid and malic aid are two of the major intermediates of the citric acid cycle to produce energy via respiration (Benkeblia et al., 2007). Heat stress typical increases respiration rates leading to an eventual decline in substrates available for energy-related reactions such as the intermediates in the citric acid cycle (Wahid et al., 2007). Elevated levels of citric acid and malic acid may indicate that ASM treated plants have higher respiratory activity to help maintain repair and defense mechanisms during prolonged heat stress. The increase accumulation of citric acid or malic acid in response to heat stress has been proposed as an important metabolic change for enhanced tolerance in a number of other metabolomics studies (Kaplan et al., 2004; Du et al., 2011; Yu et al., 2012; Jespersen et al., 2015; Li et al., 2016).

Although many less abundant sugars were increased by heat stress in both ASM and untreated plants, relatively few had significantly greater levels in ASM treated plants compared to untreated plants with the exception of the sugars arabinofuranose, gentiobiose, and gulose. Many of these sugars have the ability to act as compatible solutes to help stabilize the cell during stress events (Wang and Stutte, 1992). Other major monosaccharides such as glucose and fructose had lower concentrations in ASM treated plants than untreated plants during heat stress which may be due to changes in glycolysis and respiration rates. This shift in carbohydrate metabolism may help drive the defense and repair mechanisms needed for enhanced heat tolerance. Another metabolite which was differentially regulated between ASM and untreated plants was linolenic acid which decreased to a lower level in untreated plants during heat stress. Linolenic acid is a fatty acid which is an important membrane component, particularly in the thylakoid where it is a major target of oxidative damage (Shi et al., 2006). Greater levels of linolenic acid may represent less damage to chloroplast membranes from sustained ROS attack and increased heat tolerance.

### ASM-Responsive Proteins and Metabolites Associated with Drought Tolerance

Dehydrins are a family of proteins involved in stabilizing cells during water stress and have been demonstrated to have increased expression in *Triticum aestivum* and *Hordeum vulgare* during drought stress (Rampino et al., 2006; Tommasini et al., 2008). Dehydrin, of the measured proteins, was the only one which ASM increased during drought. Dehydrins higher accumulations in ASM treated plants under drought conditions may improve drought tolerance, as dehydrins have previously been implicated in drought tolerance in turfgrasses based on increased expression levels in *F. arundinacea* and *Lolium perenne* (Volaire et al., 1998; Jiang and Huang, 2002).

Several amino acids had higher content during drought stress when treated with ASM compared to untreated plants which included aminomalonic acid, aspartic acid, glutamic acid, glycine, and valine. Glycine, in addition to being a proteogenic amino acid is also an important precursor to a number of important secondary metabolites including porphyrins, the compatible solute glycine betaine, and the anti-oxidant glutathione (Mok and Mok, 1994; Rivero et al., 2009). Increased glycine concentrations in response to drought have been previously found in transgenic creeping bentgrasses which demonstrated improved drought tolerance (Merewitz et al., 2011). Increased glycine concentrations induced by ASM may represent a greater activation of defense pathways imparting drought tolerance involving anti-oxidant defenses and the accumulation of protective compounds. Another metabolite that had higher levels in ASM treated plants compared to untreated plants which may contribute to drought tolerance was the hydrophobic amino acid valine. Valine was previously demonstrated to have increased accumulations in *T. aestivum, Eucalyptus* spp., and *A. thaliana* in response to drought stress as a potential defense mechanism (Rizhsky et al., 2004; Bowne et al., 2012; Warren et al., 2012). Aspartic acid was also found to experience a differential response to drought stress between ASM and untreated plants with aspartic acid decreasing to a lower level in untreated plants during drought. Maintaining aspartic acid may help ASM treated plants induce drought tolerance since aspartic acid is another proteogenic amino acid which has been found to be responsive to drought stress and, as previously mentioned, aspartic acid is a precursor to many other amino acids and may play important roles in shifting plant metabolism to cope with water stress (Good and Zaplachinski, 1994; Rai, 2002; Azevedo et al., 2006).

A number of organic acids were also found to have higher accumulations during drought stress in ASM treated plants and included aconitic acid, mannonic acid, and valeric acid. Mannonic acid feeds into glucuronate metabolism which is involved in glycolysis (Wichelecki et al., 2014). Valeric acid is involved in fatty acid metabolism and the formation of precursors to the citric acid cycle (Stumpf and Barber, 1956). Although it is unclear how these metabolites may specifically influence cellular metabolism, it is known that changes in respiration can be a major factor during drought (Flexas et al., 2006). The change in accumulation of organic acids represents a shift in energy metabolism and related metabolites which, as previously discussed, may be an important factor in drought tolerance a promoted by ASM treatment.

A number of sugars increased in response to drought stress, however, only mannobiose was higher in ASM treated compared to untreated plants. Mannobiose is a disaccharide consisting of two mannose molecules. Mannose was found to be upregulated during drought in this and other metabolomic studies looking at drought stress (Du et al., 2012; Li et al., 2016) which may indicate the importance of mannose and related sugars in providing drought tolerance. The hydrolysis of more complex carbohydrates can result in increased levels of sugars in response to drought stress (Spollen and Nelson, 1994; Chaves et al., 2009) which may be a major aspect of osmotic adjustment, an important drought tolerance mechanism used to increase solute potential in the cell (Bray, 1997). Additionally ASM treated plants maintained greater levels of sitosterol compared to untreated plants during drought. Phytosterols, including sitosterols, are important membrane components which have been implicated in drought tolerance (Quartacci et al., 2002; Kumar et al., 2015). Maintaining sitosterol may be an important factor regulating membrane integrity and associated signaling pathways associated for drought tolerance.

In summary, while ASM has widely been studied in the context of promoting pathogen defense, relatively less is understood how it can prime plant systems to defend against abiotic stresses. This study demonstrated that exogenous application of ASM lead to improved heat or drought tolerance in creeping bentgrass. These heightened levels of tolerance were supported by physiological differences and also differences in the accumulation of key proteins. Metabolomics further delved into the metabolic differences between ASM and untreated plants during heat or drought. The differential accumulation of key metabolites such as amino acids, organic acids, and sugars indicate a change in metabolism which may increase abiotic stress tolerance through such mechanisms as improved energy relations, the accumulation of compatible solutes for osmotic adjustment and the stabilization of cellular constituents as well as metabolites needed for the production of important secondary metabolites involved in anti-oxidant metabolism or other stress defense mechanisms. ASM treatment resulted in distinct changes in the pattern of metabolite levels as seen with multivariate PCA analysis. A number of metabolic pathways were significant altered in both heat or drought stress conditions by treatment with ASM, including the citric acid cycle or glyoxylate metabolic pathways. However, a number of metabolite changes were unique to each stress, demonstrating the complex interaction between abiotic stresses and change to plant metabolism. Future research will look into the genetic and signaling pathways involved in the activation of these defense systems to better understand their mechanisms and how to best implement these compounds to battle abiotic stresses.

## Author Contributions

DJ performed experiments, analyzed data and wrote the manuscript. JY performed GC/MS experiments and data analysis. BH conceived and designed the experiments and wrote the manuscript.

## Conflict of Interest Statement

The authors declare that the research was conducted in the absence of any commercial or financial relationships that could be construed as a potential conflict of interest.
