# Supplementary material for: Metabolic Effects of Acibenzolar-S-Methyl for Improving Heat or Drought Stress in Creeping Bentgrass
Source: Front Plant Sci. 2017 Jul 11;8:1224. doi: 10.3389/fpls.2017.01224 (PMC5504235; doi:10.3389/fpls.2017.01224)
Supplement: Supplementary file 1 [file Table_1.DOCX]

**Supplemental table 1.** Metabolomics data for 79 identified metabolites.

|  | **Non-stress Control** | | | **Non-stress ASM** | | | **Heat stress Control** | | | **Heat stress ASM** | | | **Drought stress Control** | | | **Drought stress ASM** | | |
| --- | --- | --- | --- | --- | --- | --- | --- | --- | --- | --- | --- | --- | --- | --- | --- | --- | --- | --- |
| **Metabolite** | **Rel. Quant** | **S.E.** | **LSD** | **Rel. Quant** | **S.E.** | **LSD** | **Rel. Quant** | **S.E.** | **LSD** | **Rel. Quant** | **S.E.** | **LSD** | **Rel. Quant** | **S.E.** | **LSD** | **Rel. Quant** | **S.E.** | **LSD** |
| Acetic acid | 0.382 | 0.001 | C | 0.544 | 0.015 | A | 0.409 | 0.005 | C | 0.240 | 0.015 | D | 0.486 | 0.008 | B | 0.526 | 0.013 | AB |
| Aconitic acid | 24.531 | 0.166 | B | 31.551 | 0.442 | A | 13.262 | 0.119 | D | 18.605 | 0.207 | C | 2.492 | 0.126 | F | 3.341 | 0.196 | E |
| Alanine | 1.974 | 0.024 | F | 3.141 | 0.178 | E | 14.429 | 0.207 | A | 12.001 | 0.121 | B | 10.773 | 0.215 | C | 7.566 | 0.179 | D |
| Aminomalonic acid | 0.027 | 0.001 | D | 0.028 | 0.002 | D | 0.075 | 0.000 | A | 0.040 | 0.002 | C | 0.036 | 0.001 | C | 0.051 | 0.003 | B |
| Arabinofuranose | 0.532 | 0.011 | E | 0.584 | 0.024 | DE | 0.779 | 0.019 | B | 1.042 | 0.040 | A | 0.642 | 0.006 | CD | 0.652 | 0.020 | C |
| Arabitol | 0.095 | 0.005 | D | 0.146 | 0.011 | C | 0.710 | 0.012 | A | 0.595 | 0.004 | B | 0.575 | 0.039 | B | 0.587 | 0.026 | B |
| Asparagine | 0.090 | 0.001 | C | 0.153 | 0.001 | C | 24.029 | 0.185 | A | 21.405 | 0.127 | B | 0.185 | 0.003 | C | 0.098 | 0.008 | C |
| Aspartic acid | 0.581 | 0.016 | A | 0.482 | 0.006 | B | 0.358 | 0.008 | C | 0.580 | 0.014 | A | 0.124 | 0.001 | E | 0.168 | 0.002 | D |
| Carotenoic acid | 0.015 | 0.000 | E | 0.020 | 0.000 | D | 0.042 | 0.001 | B | 0.056 | 0.002 | A | 0.026 | 0.001 | C | 0.021 | 0.001 | D |
| Cellobiose | 0.156 | 0.003 | B | 0.153 | 0.000 | B | 0.364 | 0.029 | A | 0.400 | 0.024 | A | 0.078 | 0.001 | C | 0.063 | 0.002 | C |
| Citric acid | 1.374 | 0.021 | C | 1.101 | 0.039 | D | 3.193 | 0.086 | B | 4.105 | 0.091 | A | 0.684 | 0.011 | E | 0.811 | 0.012 | E |
| Citrulline | 0.000 | 0.000 | B | 0.000 | 0.000 | B | 0.086 | 0.011 | A | 0.090 | 0.009 | A | 0.000 | 0.000 | B | 0.000 | 0.000 | B |
| Fructose | 23.115 | 0.468 | D | 33.678 | 1.521 | B | 30.067 | 0.587 | B | 19.304 | 0.220 | E | 50.769 | 0.966 | A | 52.311 | 2.171 | A |
| GABA | 1.256 | 0.020 | E | 1.806 | 0.030 | D | 3.299 | 0.026 | A | 2.783 | 0.064 | B | 1.970 | 0.020 | C | 1.787 | 0.014 | D |
| Galactinol | 5.443 | 0.227 | D | 6.437 | 0.182 | C | 7.359 | 0.075 | B | 11.643 | 0.202 | A | 0.255 | 0.015 | E | 0.277 | 0.012 | E |
| Galactofuranoside | 0.003 | 0.000 | D | 0.003 | 0.000 | D | 0.357 | 0.006 | B | 0.460 | 0.009 | A | 0.047 | 0.018 | C | 0.037 | 0.027 | CD |
| Galactose | 1.149 | 0.026 | C | 1.644 | 0.004 | B | 2.017 | 0.046 | A | 1.763 | 0.015 | B | 1.286 | 0.016 | C | 1.256 | 0.030 | C |
| Gentiobiose | 10.866 | 0.252 | C | 21.719 | 0.303 | A | 9.375 | 0.243 | D | 15.542 | 0.308 | B | 1.564 | 0.024 | E | 1.577 | 0.024 | E |
| Glucaric acid | 0.114 | 0.003 | D | 0.094 | 0.001 | D | 0.440 | 0.006 | A | 0.359 | 0.009 | B | 0.261 | 0.005 | C | 0.260 | 0.015 | C |
| Glucitol | 0.073 | 0.001 | E | 0.134 | 0.005 | C | 0.204 | 0.007 | A | 0.185 | 0.003 | B | 0.121 | 0.005 | CD | 0.118 | 0.003 | D |
| Gluconic acid | 0.020 | 0.001 | D | 0.037 | 0.001 | B | 0.025 | 0.001 | D | 0.033 | 0.001 | B | 0.048 | 0.002 | A | 0.025 | 0.002 | D |
| Glucopyranoside | 30.759 | 0.647 | B | 36.526 | 0.291 | A | 15.489 | 0.536 | D | 22.524 | 0.240 | C | 8.277 | 0.159 | E | 8.118 | 0.325 | E |
| Glucopyranuronic acid | 0.066 | 0.001 | C | 0.066 | 0.002 | C | 0.268 | 0.012 | A | 0.231 | 0.006 | A | 0.134 | 0.004 | B | 0.123 | 0.008 | B |
| Glucose | 4.078 | 0.223 | E | 6.587 | 0.434 | D | 15.495 | 0.654 | A | 13.883 | 0.358 | B | 9.908 | 0.086 | C | 9.495 | 0.103 | C |
| Glucuronic acid | 0.054 | 0.002 | C | 0.077 | 0.001 | C | 0.417 | 0.049 | A | 0.449 | 0.019 | A | 0.160 | 0.005 | B | 0.146 | 0.001 | B |
| Glutamic acid | 5.602 | 0.166 | A | 4.115 | 0.057 | C | 4.686 | 0.147 | B | 5.982 | 0.050 | A | 0.706 | 0.039 | E | 1.279 | 0.027 | D |
| Glutamine | 0.044 | 0.002 | D | 0.063 | 0.003 | D | 1.791 | 0.028 | B | 1.972 | 0.008 | A | 0.322 | 0.011 | C | 0.244 | 0.022 | C |
| Glutaric acid | 0.012 | 0.000 | C | 0.017 | 0.000 | C | 0.271 | 0.007 | A | 0.248 | 0.015 | A | 0.122 | 0.003 | B | 0.121 | 0.004 | B |
| Glyceric Acid | 2.760 | 0.170 | B | 3.227 | 0.188 | A | 0.374 | 0.010 | C | 0.500 | 0.004 | C | 0.415 | 0.008 | C | 0.453 | 0.020 | C |
| Glycerol | 5.456 | 0.048 | BC | 7.355 | 0.251 | A | 5.031 | 0.065 | C | 4.348 | 0.021 | D | 5.494 | 0.104 | B | 5.829 | 0.066 | B |
| Glycine | 2.802 | 0.132 | E | 4.411 | 0.064 | C | 11.911 | 0.550 | B | 13.186 | 0.211 | A | 3.596 | 0.190 | D | 4.688 | 0.196 | C |
| Gulose | 0.074 | 0.003 | E | 0.087 | 0.002 | E | 0.324 | 0.006 | B | 0.350 | 0.007 | A | 0.140 | 0.003 | C | 0.106 | 0.002 | D |
| Histidine | 0.309 | 0.005 | BC | 0.382 | 0.024 | A | 0.352 | 0.006 | AB | 0.289 | 0.008 | C | 0.114 | 0.004 | D | 0.103 | 0.001 | D |
| Homoserine | 0.033 | 0.001 | C | 0.020 | 0.001 | C | 0.114 | 0.013 | A | 0.090 | 0.010 | AB | 0.046 | 0.008 | BC | 0.032 | 0.003 | C |
| Inositol | 0.000 | 0.000 | C | 0.000 | 0.000 | C | 0.056 | 0.001 | A | 0.045 | 0.002 | B | 0.053 | 0.002 | A | 0.000 | 0.000 | C |
| Isoleucine | 0.370 | 0.028 | D | 0.480 | 0.005 | D | 2.880 | 0.067 | C | 3.410 | 0.213 | B | 3.886 | 0.164 | A | 3.567 | 0.095 | AB |
| Leucine | 0.606 | 0.046 | C | 0.781 | 0.079 | C | 2.599 | 0.230 | B | 2.467 | 0.167 | B | 3.230 | 0.079 | A | 3.345 | 0.231 | A |
| Linolenic acid | 0.329 | 0.007 | B | 0.449 | 0.014 | A | 0.031 | 0.001 | E | 0.124 | 0.007 | D | 0.160 | 0.004 | C | 0.150 | 0.002 | C |
| Lysine | 0.048 | 0.002 | C | 0.057 | 0.003 | C | 0.867 | 0.003 | B | 0.997 | 0.035 | A | 0.089 | 0.000 | C | 0.084 | 0.001 | C |
| Lyxopyranoside | 0.000 | 0.000 | C | 0.000 | 0.000 | C | 0.130 | 0.007 | A | 0.140 | 0.001 | A | 0.017 | 0.000 | B | 0.000 | 0.000 | C |
| Lyxose | 0.644 | 0.027 | C | 0.643 | 0.032 | C | 1.060 | 0.065 | B | 1.056 | 0.015 | B | 1.206 | 0.058 | A | 1.232 | 0.071 | A |
| Maleic acid | 0.000 | 0.000 | D | 0.002 | 0.000 | C | 0.003 | 0.000 | C | 0.003 | 0.000 | C | 0.022 | 0.001 | A | 0.015 | 0.000 | B |
| Malic acid | 4.328 | 0.178 | F | 5.515 | 0.132 | E | 9.907 | 0.190 | D | 10.774 | 0.183 | C | 18.545 | 0.114 | A | 14.588 | 0.102 | B |
| Malonic acid | 0.022 | 0.006 | A | 0.017 | 0.001 | A | 0.000 | 0.000 | B | 0.000 | 0.000 | B | 0.000 | 0.000 | B | 0.000 | 0.000 | B |
| Maltose | 0.429 | 0.017 | B | 0.620 | 0.004 | A | 0.482 | 0.030 | B | 0.463 | 0.018 | B | 0.485 | 0.012 | B | 0.456 | 0.011 | B |
| Mannitol | 0.045 | 0.002 | D | 0.062 | 0.002 | D | 0.317 | 0.010 | A | 0.252 | 0.016 | B | 0.125 | 0.002 | C | 0.145 | 0.003 | C |
| Mannobiose | 0.263 | 0.016 | C | 0.313 | 0.004 | BC | 0.262 | 0.005 | C | 0.172 | 0.003 | D | 0.368 | 0.013 | B | 0.502 | 0.003 | A |
| Mannonic acid | 0.113 | 0.006 | C | 0.108 | 0.003 | C | 0.058 | 0.002 | D | 0.069 | 0.002 | D | 0.152 | 0.002 | B | 0.204 | 0.002 | A |
| Mannose | 1.220 | 0.063 | D | 1.438 | 0.064 | D | 7.487 | 0.628 | A | 7.611 | 0.380 | A | 6.708 | 0.369 | B | 3.664 | 0.213 | C |
| Methionine | 0.000 | 0.000 | C | 0.000 | 0.000 | C | 0.345 | 0.020 | A | 0.360 | 0.009 | A | 0.041 | 0.003 | B | 0.038 | 0.001 | B |
| Myo-Inositol | 2.656 | 0.050 | C | 4.248 | 0.067 | B | 11.506 | 0.180 | A | 11.985 | 0.277 | A | 1.091 | 0.065 | D | 1.085 | 0.035 | D |
| Norvaline | 0.033 | 0.001 | C | 0.033 | 0.002 | C | 0.115 | 0.001 | A | 0.117 | 0.004 | A | 0.052 | 0.003 | B | 0.052 | 0.002 | B |
| Oxalic acid | 5.664 | 0.118 | A | 5.491 | 0.104 | A | 4.468 | 0.160 | B | 3.173 | 0.066 | C | 5.170 | 0.084 | A | 5.489 | 0.140 | A |
| Phosphonic acid | 0.053 | 0.006 | DE | 0.068 | 0.003 | C | 0.151 | 0.004 | B | 0.187 | 0.003 | A | 0.044 | 0.003 | EF | 0.038 | 0.002 | F |
| Proline | 0.111 | 0.004 | E | 0.155 | 0.001 | E | 10.306 | 0.077 | C | 5.446 | 0.232 | D | 31.331 | 0.374 | A | 26.154 | 0.743 | B |
| Propenoic acid | 0.173 | 0.005 | D | 0.210 | 0.001 | D | 1.268 | 0.034 | A | 1.130 | 0.035 | B | 0.358 | 0.026 | C | 0.373 | 0.012 | C |
| Psicose | 0.146 | 0.008 | D | 0.817 | 0.036 | A | 0.869 | 0.034 | A | 0.863 | 0.054 | A | 0.705 | 0.034 | B | 0.208 | 0.012 | C |
| pyruvic acid | 0.328 | 0.009 | ABC | 0.225 | 0.015 | E | 0.364 | 0.010 | AB | 0.271 | 0.015 | CDE | 0.313 | 0.011 | BCD | 0.233 | 0.014 | DE |
| Serine | 2.840 | 0.054 | D | 4.559 | 0.292 | C | 14.117 | 0.693 | B | 15.329 | 0.290 | A | 1.306 | 0.008 | E | 1.486 | 0.038 | E |
| Shikimic acid | 8.287 | 0.206 | B | 10.422 | 0.216 | A | 1.996 | 0.036 | D | 1.696 | 0.004 | D | 2.718 | 0.137 | C | 2.539 | 0.030 | C |
| Sitosterol | 0.604 | 0.005 | A | 0.580 | 0.007 | A | 0.086 | 0.002 | E | 0.339 | 0.018 | C | 0.261 | 0.006 | D | 0.527 | 0.011 | B |
| Sucrose | 56.021 | 1.325 | C | 67.021 | 1.282 | B | 72.565 | 1.298 | AB | 77.616 | 1.449 | A | 75.064 | 1.212 | A | 77.004 | 2.137 | A |
| Talose | 19.491 | 1.154 | C | 26.557 | 2.552 | B | 20.868 | 1.139 | C | 15.246 | 0.663 | D | 34.567 | 3.713 | A | 35.777 | 1.175 | A |
| Threonic acid | 0.071 | 0.001 | D | 0.102 | 0.007 | C | 0.056 | 0.001 | E | 0.034 | 0.002 | F | 0.239 | 0.005 | A | 0.223 | 0.005 | B |
| Threonine | 0.425 | 0.027 | E | 0.583 | 0.029 | DE | 1.884 | 0.030 | B | 2.284 | 0.067 | A | 0.722 | 0.026 | CD | 0.830 | 0.012 | C |
| Trehalose | 0.051 | 0.001 | B | 0.063 | 0.001 | A | 0.026 | 0.001 | C | 0.031 | 0.002 | C | 0.063 | 0.003 | A | 0.054 | 0.001 | AB |
| Tryptophan | 0.350 | 0.009 | D | 0.218 | 0.005 | D | 7.713 | 0.104 | B | 6.641 | 0.233 | C | 8.287 | 0.110 | A | 7.585 | 0.051 | B |
| Turanose | 31.128 | 0.809 | C | 28.516 | 0.586 | C | 38.860 | 0.223 | B | 42.524 | 2.208 | AB | 45.524 | 1.188 | A | 41.945 | 1.255 | AB |
| Tyrosine | 0.272 | 0.005 | C | 0.344 | 0.025 | C | 2.022 | 0.018 | A | 2.232 | 0.032 | A | 0.965 | 0.037 | B | 0.921 | 0.018 | B |
| Valeric acid | 0.049 | 0.002 | D | 0.066 | 0.001 | C | 0.105 | 0.003 | A | 0.094 | 0.005 | B | 0.074 | 0.001 | C | 0.091 | 0.002 | B |
| Valine | 0.270 | 0.019 | C | 0.282 | 0.009 | C | 1.757 | 0.030 | A | 1.816 | 0.040 | A | 1.358 | 0.045 | B | 1.628 | 0.097 | A |
| Xylulose | 0.043 | 0.001 | C | 0.058 | 0.002 | C | 1.014 | 0.020 | A | 1.105 | 0.035 | A | 0.253 | 0.008 | B | 0.234 | 0.008 | B |

**Rel. Quant – Relative quantities**

**S.E. – Standard error**

**LSD – LSD grouping at p < 0.05**
